# Interrogation of the intersubunit interface of the open Hv1 proton channel with a probe of allosteric coupling

**DOI:** 10.1038/srep14077

**Published:** 2015-09-14

**Authors:** Liang Hong, Vikrant Singh, Heike Wulff, Francesco Tombola

**Affiliations:** 1 Department of Physiology and Biophysics, University of California, Irvine, CA 92697, USA; 2 Department of Pharmacology, University of California, Davis, CA 95616, USA

## Abstract

The Hv1 voltage-gated proton channel is a dimeric complex consisting of two voltage-sensing domains (VSDs), each containing a gated proton permeation pathway. Dimerization is controlled by a cytoplasmic coiled-coil domain. The transitions from the closed to the open state in the two VSDs are known to occur cooperatively; however, the underlying mechanism is poorly understood. Intersubunit interfaces play a critical role in allosteric processes; but, such interfaces have not been determined in the open Hv1 channel. Here we show that 2-guanidinothiazole derivatives block the two Hv1 VSDs in a cooperative way, and use one of the compounds as a probe of allosteric coupling between open subunits. We find that the extracellular ends of the first transmembrane segments of the VSDs form the intersubunit interface that mediates coupling between binding sites, while the coiled-coil domain does not directly participate in the process. We also find strong evidence that the channel’s proton selectivity filter controls blocker binding cooperativity.

Voltage-gated proton channels serve important functions in various organisms, from phytoplankton to humans^1^. In most cells, these channels mediate proton efflux at the plasma membrane and regulate the activity of the NADPH oxidase. The only known voltage-gated proton channel in humans is Hv1, the product of the *HVCN1* gene^2^,^3^. Hv1 (a.k.a. VSOP) has been shown to play a role in B-cell proliferation^4^, in the production of reactive oxygen species by the innate immune system^5^,^6^,^7^,^8^, in sperm cell motility^9^, and in pH regulation of the airway surface liquid^10^. The channel is over-expressed in several types of cancer, such as B-cell malignancies^4^,^11^, and breast and colorectal cancers^12^,^13^. Excessive Hv1 activity was found to increase the metastatic potential of cancer cells^11^,^12^. In the brain, Hv1 is expressed by microglia and its activity was shown to worsen brain damage in a model of ischemic stroke^8^.

The Hv1 protein contains a voltage-sensing domain (VSD) consisting of four membrane-spanning segments, named S1 through S4^14^. The VSD is similar to the corresponding domain of voltage-gated Na^+^, K^+^, and Ca^2+^ channels, and voltage-sensitive phosphatases, such as CiVSP from *Ciona intestinalis*^15^. In these other proteins, the C-terminal end of S4 is connected to an effector module, either a pore domain or an enzyme. In Hv1, S4 is connected to a coiled-coil domain (CCD) located on the cytoplasmic side of the membrane. The channel is a dimeric complex made of two VSDs each containing a gated proton permeation pathway^16^,^17^,^18^. The two Hv1 subunits were found to open cooperatively^19^,^20^,^21^,^22^ indicating that allosteric coupling and intersubunit interactions play an important role in the gating process. The interface between subunits within the coiled-coil domain is well defined, as two crystal structures of the isolated domain are available^22^,^23^. On the other hand, the interface between VSDs within the membrane is not well understood. The crystal structure of the Hv1-CiVSP chimeric protein^24^ does not provide information about this interface due to the trimeric organization of the crystallized channel complex, likely induced by the replacement of the native Hv1 CCD with the yeast leucine-zipper GCN4^24^.

A recent study of the subunit organization of the Hv1 channel concluded that the two S4 helices transition into the CCD without major interruptions of secondary structure, producing long helices that start in the membrane and project into the cytoplasm^25^. Based on cysteine cross-linking analysis, the study proposed that the Hv1 VSDs contact each other along the S4 segments. However, alternative interfaces between VSDs have been proposed by other studies. These interfaces include the outer ends of the S1 segments^17^,^21^,^26^ and the S2 segments^21^. One possible cause for the conflicting results of these studies is that allosteric coupling between VSDs was examined in relation to the gating process^21^,^25^,^26^, which depends on both closed and open states, and the interface between VSDs could change conformation in different states.

Here we find that 2-guanidinothiazoles inhibit the Hv1 channel by binding cooperatively to the two open VSDs, and use one of these compounds, 2-guanidinobenzothiazole (GBTA), to probe the interface between subunits in the open state. We find that the GBTA binding curve is well described by a quantitative model in which the binding of the inhibitor to one subunit induces an increase in binding affinity of the neighboring subunit. We also find that residue D112, the channel’s selectivity filter^27^,^28^ and part of the binding site for guanidine derivatives^29^, controls GBTA binding cooperativity. We show that the cooperative binding is maintained in Hv1 dimers in which the CCD is uncoupled from the S4 segment indicating that the intersubunit interface in the CCD does not directly mediate allosteric coupling between GBTA binding sites. In contrast, we find that the S1 segments are part of the interface between subunits and propose an arrangement of the neighboring VSDs in which the extracellular ends of the S4 helices move away from the center of the dimer to allow the S1 segments to interact in the open state.

## Results

### Hv1 channel inhibition by 2-guanidino-thiazole compounds

**Figure 1:**
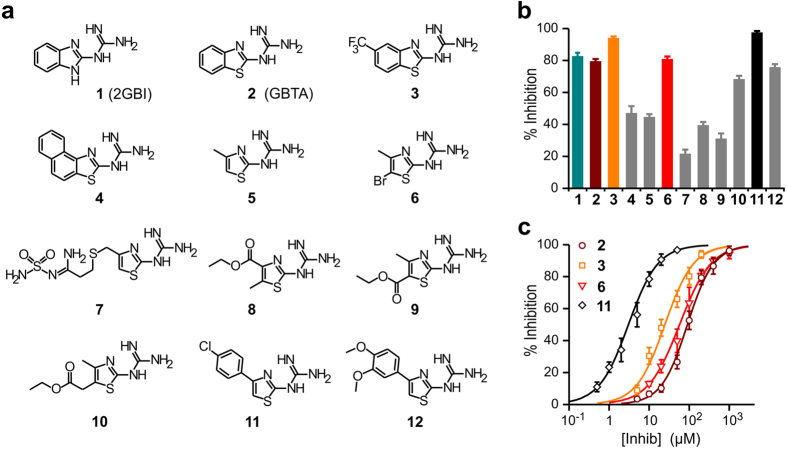
Hv1 inhibition by guanidinothiazole derivatives. (**a**) Tested compounds: [1] reference Hv1 inhibitor 2-guanidino-benzimidazole (2GBI). [2] 2-guanidino-benzothiazole (GBTA), [3] (5-trifluoromethyl-1,3-benzothiazol-2-yl)guanidine, [4] naphtho[1,2*-d*][1,3]thiazol-2-yl-guanidine, [5] (4-methyl-1,3-thiazol-2-yl)guanidine, [6] (5-bromo-4-methyl-1,3-thiazol-2-yl)guanidine, [7] famotidine, [8] ethyl 2-guanidino-5-methyl-1,3-thiazole-4-carboxylate, [9] ethyl 2-guanidino-4-methyl-1,3-thiazole-5-carboxylate, [10] ethyl (2-guanidino-4-methyl-1,3-thiazol-5-yl)acetate, [11] 1-[4-(4-chlorophenyl)-1,3-thiazol-2-yl]guanidine, [12] 1-[4-(3,4-dimethoxyphenyl)-1,3-thiazol-2-yl]guanidine. (**b**) Inhibition of human Hv1 activity by the indicated guanidinothiazoles and reference compound 2GBI (teal column). Hv1 proton currents were measured in inside out patches from *Xenopus* oocytes in response to depolarizations to +120 mV from a holding potential of −80 mV. Each inhibitor was added to the bath solution at a concentration of 200 μM. pH_i_ = pH_o_ = 6.0. Data are means ± S.E.M. (n ≥ 4). (**c)** Concentration dependences of inhibition of human Hv1 by compounds [2], [3], [6], and [11]. Each point represents the average inhibition from 3 to 15 measurements ± S.D. Curve lines are Hill fits used to obtain the apparent K_d_ values reported in Supplementary Table 1. Hill coefficients were determined from fits reported in Supplementary Fig. 1: *h*(1) = 0.975 ± 0.024 *h*(2) = 1.306 ± 0.033, *h*(3) = 1.25 ± 0.07, *h*(6) = 1.109 ± 0.040, *h*(11) = 1.179 ± 0.036 (see methods).

**Figure 2:**
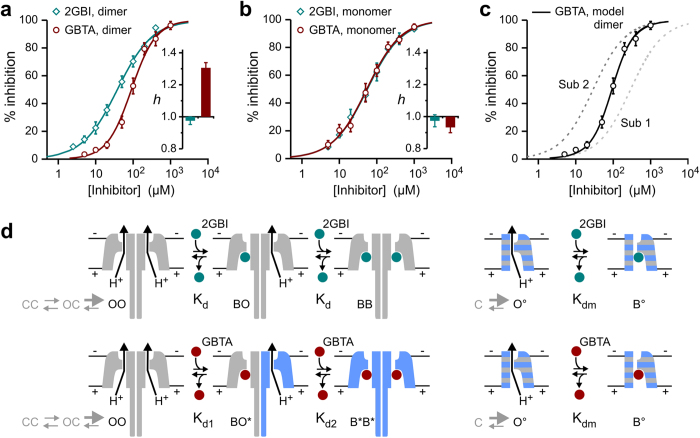
Guanidinothiazole derivatives bind to the Hv1 dimer in a cooperative way. (**a,b**) Concentration dependences of inhibition of dimeric and monomeric Hv1 by compounds 2GBI and GBTA. Each point represents the average inhibition from 3 to 8 measurements ± S.D. Curve lines are Hill fits. Hill coefficients (*h*) shown in inset histograms were determined from fits reported in Supplementary Figs 3 and 4. The concentration response for GBTA shown in (**a**) is the same as in Fig. 1c. For apparent K_d_ values see Supplementary Table 1. (**c**) Modeling of cooperative binding of GBTA to dimeric Hv1. Solid black line represents the fit of the experimental data by equation (6), which describes the binding model shown in (**d**). Dotted lines marked as Sub 1 and Sub 2 represent the bimolecular association-dissociation equilibrium curves for the first and second binding events, respectively (Sub 1: OO + B ⇄ BO*, K_d1_ = 290 ± 70 μM; Sub 2: BO* + B ⇄ B*O*, K_d2_ = 29.3 ± 2.5 μM). (**d)** Schematic representation of the proposed mechanism of Hv1 block. In the case of GBTA, binding to one open subunit, increases the affinity of the neighboring open subunit (K_d2_ < K_d1_). Subunits are gray when in the low affinity state and blue when in the high affinity state. For the binding of GBTA to the monomerized Hv1, the dissociation constant of the reaction: O° + B ⇄ B° has a value between K_d1_ and K_d2_, but closer to the latter (gray-blue stripes indicate intermediate affinity).

Small-molecule inhibitors of Hv1 could find applications as anticancer drugs and neuroprotective agents. However, few compounds have been shown to inhibit the channel thus far^30^,^31^,^32^,^33^. Among these, 2-guanidinobenzimidazole (2GBI, compound [1] in Fig. 1a) and its derivatives were found to block proton permeation through the channel’s VSDs^29^,^32^. The binding of this class of compounds is believed to occur independently in the two open subunits^32^. 2-Guanidino-benzothiazole (GBTA, compound [2] in Fig. 1a) was previously shown^32^ to inhibit Hv1 almost as effectively as 2GBI when tested at a concentration of 200 μM (Fig.1b). We examined other thiazole derivatives and found that some of them inhibited the channel with similar or higher potency compared to GBTA (Fig. 1 and Supplementary text). We determined the concentration response curves of four thiazole derivatives (GBTA and compounds [3], [6], and [11], Fig. 1c) and found that they were steeper than the concentration response of 2GBI. The Hill coefficients (*h*) for the thiazole derivatives ranged from 1.109 ± 0.040 to 1.306 ± 0.033 (Fig.1c and Supplementary Fig. 1). In contrast, the Hill coefficient for 2GBI was 0.975 ± 0.024 29, Fig. 2a and Supplementary Fig. 1). Hill coefficients higher than 1 are indicative of binding cooperativity. Because each Hv1 subunit has its own binding site for the inhibitor^29^,^32^, we reasoned that the binding of the thiazole derivative to one subunit could enhance the binding of a second molecule of inhibitor to the adjacent subunit. GBTA was the tested compound with the highest Hill coefficient. So, we chose this compound to further investigate the mechanism of binding cooperativity and used 2GBI as reference negative control.

### Mechanism of GBTA binding to Hv1

The Hv1 channel can be monomerized by replacing its N- and C-terminal cytoplasmic domains with the corresponding parts of the *Ciona Intestinalis* voltage-sensitive phosphatase CiVSP (Hv1NC_CiVSP_)^18^,^34^. We measured the concentration-dependences of inhibition of monomeric Hv1 by GBTA and 2GBI and compared them to the concentration-dependences of inhibition of the dimeric channel (wild type) (Fig. 2a,b). We found that the difference in binding cooperativity between the two compounds was abolished in monomeric Hv1, supporting the interpretation that the binding of GBTA to one subunit increases the affinity of the other subunit for the inhibitor. We reasoned that GBTA binding to one Hv1 subunit could cause a rearrangement in the binding site (induced fit^35^) that would trigger a rearrangement in the empty binding site of the neighboring subunit resulting in an increase in binding affinity.

To describe quantitatively the cooperative binding of GBTA to the channel, we used a model in which either subunit can bind the first molecule of inhibitor following a bimolecular reaction with a dissociation constant K_d1_ (Fig. 2c, Sub 1, OO + B ⇆ BO*). The binding causes the channel to adopt a state in which the remaining empty subunit binds the inhibitor following a distinct bimolecular reaction with dissociation constant K_d2_, where K_d2_ < K_d1_ (Fig. 2c, Sub 2, BO* + B ⇆ B*B*). Once the second molecule of inhibitor is bound, both subunits have dissociation constant K_d2_ (Fig. 2d). Channel inhibition was measured under depolarized conditions (+120 mV). Channel species with one open and one closed subunit (see transition CC ⇄ OC ⇄ OO in Fig. 2d) were previously found to provide negligible contribution to the Hv1 current under these conditions^19^,^20^, and for this reason they were not included in the binding model.

The solid black line in Fig. 2c is the fit of the experimental concentration response curve by the model equation, yielding a K_d1_ of ~290 μM and a K_d2_ of ~29 μM (Methods section, equation (6)). The model also describes the binding of 2GBI, where K_d2_ ≈ K_d1_ = K_d_ (Fig. 2d). In the monomerized Hv1, there is only one binding site and one K_dm_ (O° + B ⇆ B°). In the case of GBTA, K_dm_ turned out to be ~54 μM, which is more similar to K_d2_ than to K_d1_ (Fig. 2d). In other words, the binding site of the monomer is in a configuration (O°) more similar to the high affinity state (BO*) than the low affinity state (OO) of the dimer, a result most likely due to the elimination of the interface between subunits in the monomer.

As previously shown for 2GBI^32^, we found that GBTA inhibits the Hv1 current by blocking the channels when they are open rather than by making the channels harder to open (Supplementary Fig. 2a,b,d). 2GBI is also known to induce slowly decaying tail currents in response to membrane repolarization^32^, but this phenomenon was not observed with GBTA (Supplementary Fig. 2c). In the case of Hv1 deactivation in the presence of 2GBI, the gate in each subunit cannot close until the blocker leaves the binding site (“foot in the door” mechanism) and 2GBI unbinding is slower than gate closing. If one Hv1 subunit becomes unblocked and closes while the adjacent subunit is still blocked, then the unbinding of the remaining 2GBI molecule becomes much slower (blocker trapping). Long-lived channel species with only one blocked subunit transiently conduct protons before closing and provide an important contribution to the slowly decaying tail currents^32^.

The finding that the decay of Hv1 tail currents is not significantly slowed down in the presence of GBTA (Supplementary Fig. 2c) is consistent with the proposed mechanism of cooperative binding for this blocker (Fig. 2d). Once a molecule of GBTA unbinds from an Hv1 subunit, the affinity for the blocker to the adjacent subunits drops ~10 fold, favoring the unbinding process. This means that there is a much higher chance for the second molecule of GBTA to unbind before it can be trapped in the channel. The long-lived channel species with only one blocked subunit generated in the presence of GBTA are expected to be much less abundant than in the case of 2GBI, leading to a faster decay of the tail currents.

### GBTA binding cooperativity is mediated by D112 in the binding site

**Figure 3:**
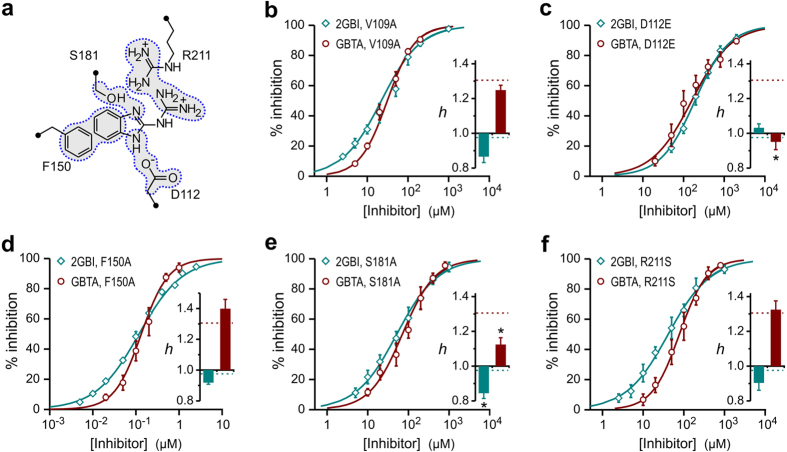
GBTA binding cooperativity is modified by perturbations of the binding site. (**a**) Hv1 residues proposed to be involved in the binding of guanidine derivatives. Dotted blue curves encircle side chains previously proposed to interact with different parts of compound 2GBI^29^. (**b**–**f)** Concentration dependences of inhibition of the indicated Hv1 mutants by compounds 2GBI (teal) and GBTA (dark red). Each point represents the average inhibition from 3 to 12 measurements ± S.D. V109 served as a negative control. Curve lines are Hill fits used to obtain apparent K_d_ values (see Supplementary Table 1). Hill coefficients (*h*) shown in inset histograms were determined from fits reported in Supplementary Figs 3 and 4. Reference *h* values for Hv1 WT are shown as dotted lines. Asterisk indicates statistically significant difference between mutant and WT channel (p < 0.05/14, see methods).

In order for GBTA to bind cooperatively to the two Hv1 subunits, the binding sites must be allosterically coupled. Each binding site must be able to: 1) trigger the chain of events that communicate to the neighboring subunit that the inhibitor is bound, and 2) mediate the switch from low affinity to high affinity binding. We reasoned that if specific residues within the binding site contribute to the cooperative process, their mutation should change the Hill coefficient of the concentration response curve of Hv1 inhibition by GBTA. Residues D112, F150, S181, and R211 were previously shown to be part of the binding environment of 2GBI^29^ and we assumed that they would be similarly involved in the binding of GBTA (Fig. 3A). We measured the concentration response curves of inhibition of mutant channels D112E, F150A, S181A, and R211S (Fig. 3) by GBTA and compared their Hill coefficients to the coefficient of Hv1 wild type, using 2GBI as a reference. Residue V109 is on the same face of the S1 segment as D112 and one helical turn more intracellular. Since V109 does not participate in the binding of 2GBI^29^, we used the V109A mutant as a control (Fig. 3b).

We found that the D112E mutation significantly reduced the Hill coefficient compared to wild type for GBTA (p < 0.05/14 see methods), but not for 2GBI (Fig. 3c and Supplementary Figs 3 & 4). The S181A mutation caused significant changes in Hill coefficients for both GBTA and 2GBI (p < 0.05/14, Fig. 3e and Supplementary Figs 3 & 4), whereas mutations V109A, F150A, and R211S did not cause significant changes in the Hill coefficients (Fig. 3 and Supplementary Figs 3 & 4). We conclude that GBTA binding cooperativity is abolished in D112E channels and partially reduced in S181A channels. However, the finding that the S181A mutation reduces the Hill coefficient for 2GBI binding to a value significantly lower than 1 with minor effects on potency (Fig. 3e) suggests that this mutation generates some negative cooperativity between the binding sites. In support of negative cooperativity, we found that the change in Hill coefficient for 2GBI inhibition induced by the S181A mutation was smaller (and no longer statistically significant) in the monomer background (Supplementary Fig. 5).

### Testing the model for GBTA binding cooperativity in a linked dimer

**Figure 4:**
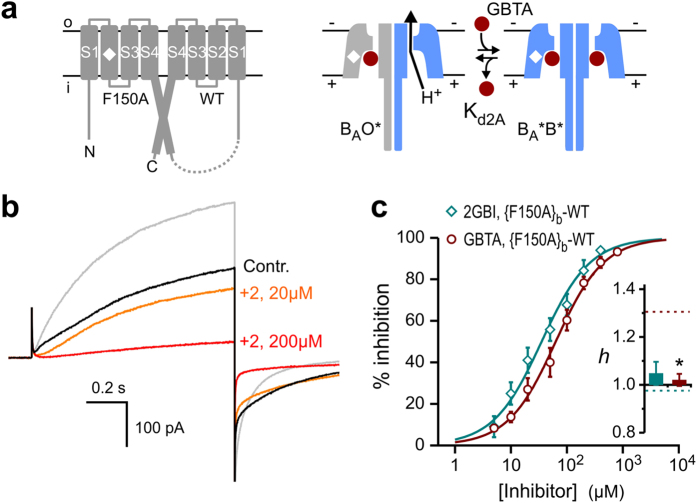
Binding of GBTA to blocked Hv1 hemichannels. (**a**) Schematics of the F150A-WT linked dimer used to generate blocked hemichannels ({F150A}_b_-WT/B_A_O*). White diamond shows position of mutation. The affinity of the F150A subunit is expected to be higher than the WT subunit in both the B_A_O* and B_A_*B* states. (**b)** Proton current from F150A-WT channels measured in response to a change in membrane potential from −80 mV to +120 mV. pH_i_ = pH_o_ = 6.0. Gray trace represents the current measured in the absence of inhibitor. Black trace (Control) represents the current measured after addition of 2 μM GBTA. At this concentration, the WT subunit is not significantly inhibited, while the F150A subunit is almost completely bound to GBTA ({F150A}_b_-WT). Further increase in GBTA concentration causes the block of the WT subunit (orange and red traces). (**c)** Concentration dependences of inhibition of the {F150A}_b_-WT dimer (with inhibitor pre-bound to the F150A subunit). Each point represents the average inhibition from 3 to 7 measurements ± S.D. Curve lines are Hill fits used to obtain the apparent K_d_ values reported in Supplementary Table 1. Hill coefficients shown in inset histogram were determined from fits reported in Supplementary Figs 3 and 4. Reference *h* values for Hv1 WT are shown as dotted lines. Asterisk indicates statistically significant difference compared to WT dimeric Hv1 (p < 0.05/14, see methods).

The finding that the Hill coefficient for GBTA binding is higher than 1 in the Hv1 dimer and becomes ~1 in the monomeric channel (Fig. 2b) is consistent with the existence of allosteric interactions between binding sites in the two subunits. If this is the case, the Hill coefficient for GBTA binding to a dimer in which the blocker is already bound to one subunit should be ~1. We tested this prediction in the Hv1 F150A-WT linked dimer (Fig. 4a) in which the F150A subunit has an affinity for GBTA more than 2 orders of magnitude higher than the WT subunit (see Figs 1c and 3d). We then measured the concentration response curve of inhibition of the WT subunit in the presence of a basal concentration of 2μM GBTA. At this concentration, the blocker is expected to bind to ~99% of the F150A subunit and <2% of the WT subunit, thus producing {F150A}_b_-WT (or B_A_O*) blocked hemichannels (Fig. 4a). The reduction in proton current measured after addition of 2μM GBTA to F150A-WT channels (Fig. 4b) was consistent with the inhibition of the F150A subunit. The concentration response curve of the blocked hemichannels yielded a Hill coefficient very close to 1 (Fig. 4c), confirming that GBTA binding cooperativity arises from allosteric coupling between binding sites on adjacent subunits rather than coupling between intrasubunit binding sites.

### Allosteric coupling between GBTA binding sites is not mediated by the coiled-coil domain

**Figure 5:**
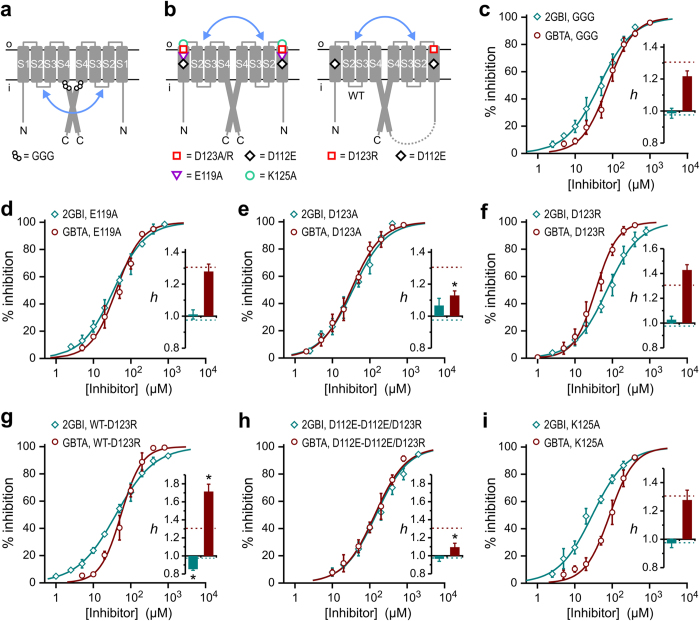
Exploring the roles of CCD and S1 in coupling between open subunits. (**a**) Schematics of an Hv1 dimer with a triple glycine mutation at the inner end of S4 designed to disrupt intersubunit coupling mediated by the cytoplasmic coiled-coil domain (blue arrow). (**b**) Schematics of Hv1 dimer and linked dimer bearing the indicated mutations designed to test the involvement of the S1 segment in the coupling between subunits (blue arrow). (**c–h**) Concentration dependences of inhibition of the indicated constructs by 2GBI (teal) and GBTA (dark red). Each point represents the average inhibition from 3 to 10 measurements ± S.D. Curve lines are Hill fits used to obtain apparent K_d_ values (see Supplementary Table 1). Hill coefficients in inset histograms were determined as described in the methods section (see Supplementary Figs 3 and 4). Reference *h* values for Hv1 WT are shown as dotted lines. Asterisk indicates statistically significant difference between mutant and WT channel (p < 0.05/14, see methods).

Since Hv1 inhibition by GBTA is measured in open channels, we reasoned that GBTA binding cooperativity could be used to investigate the mechanism of intersubunit coupling in the open state. The two Hv1 subunits were previously found to gate cooperatively^19^,^20^,^21^,^22^ and the intersubunit coupling involved in gating was proposed to be mediated by the cytoplasmic coiled-coil domain (CCD)^22^,^25^. Hence, we asked whether the CCD is also involved in the coupling between GBTA binding sites in the open state. A triple glycine mutation at the interface between the S4 transmembrane segment and the coiled-coil domain was shown in an earlier study to uncouple the domain from the rest of the channel while maintaining dimerization intact^22^. So, we tested the effect of mutations V220G, K221G, and T222G (Fig. 5a, GGG mutant) on GBTA binding cooperativity. We found a small, statistically not significant (p > 0.05), reduction in Hill coefficient of GBTA binding to the GGG channel compared to wild type (Fig. 5c), indicating that, despite its importance in keeping the two subunits together, the CCD does not directly mediate the intersubunit allosteric coupling between GBTA binding sites.

### Binding sites in the two subunits are allosterically coupled via S1 segments

Since we excluded the CCD as a direct mediator of cooperative binding of GBTA, we asked whether interactions between VSDs participate in the allosteric coupling between binding sites. We found that GBTA binding cooperativity is abolished by a conservative mutation of residue D112 (Fig. 3c) located in the S1 transmembrane segment. S1 contains two other negatively charged residues, E119 and D123, on the same side of the helix as D112, but closer to the outer end of the segment^24^. Earlier molecular dynamics simulations suggested that these residues are connected to D112 via water wires^36^,^37^. So, we tested whether E119 and D123 are involved in the allosteric coupling between GBTA binding sites (Fig. 5b). As a negative control, we tested the conserved positively charged position K125, which is located in proximity of D123, but on the opposite side of the S1 helix^24^ (Fig. 5b).

We measured concentration dependences of inhibition of E119A, D123A, and K125A channels by 2GBI and GBTA, and found that the E119A and K125A mutations did not change significantly the Hill coefficient for either inhibitor compared to wild type (p > 0.05, Fig. 5d,i). On the other end, mutation D123A significantly reduced the Hill coefficient for GBTA (p < 0.05/14, Fig. 5e and Supplementary Fig. 4), suggesting that electrostatic interactions between charged residues are involved in allosteric coupling between GBTA binding sites. We further investigated this possibility by examining the effect of a positive charge at position 123 on binding cooperativity. The D123R mutation increased the Hill coefficient for channel inhibition by GBTA, compared to wild type (Fig. 5f). But, the extent of the increase was small and did not meet our criterion for statistical significance (p > 0.05/14).

Since neutralizing the charge at position 123 on both subunits resulted in a strong change in GBTA binding cooperativity, while inverting the charge on both subunits produced only a small effect, we extended our analysis to channels in which only one subunit had the inverted charge. We generated Hv1 linked-dimers with the D123R substitution in the C-terminal subunit (Fig. 5b), and measured concentration responses of inhibition for GBTA and 2GBI. We found that the Hill coefficient for GBTA binding to WT-D123R channels was significantly higher than the Hill coefficient for wild type Hv1 (p < 0.05/14, Fig. 5g and Supplementary Fig. 4), indicating a strengthening in allosteric coupling between binding sites (increase in positive cooperativity for GBTA binding). Conversely, the Hill coefficient for 2GBI binding to WT-D123R channels was significantly lower than the Hill coefficient of wild type Hv1 (p < 0.05/14, Fig. 5g and Supplementary Fig. 3). The reduction of the coefficient to a value lower than 1 suggests that the manipulation of the allosteric coupling between binding sites can result in an increase in negative cooperativity for 2GBI binding.

We also found that the D112E mutation abolished the increase in Hill coefficient for GBTA binding produced by the WT-D123R background (Fig. 5b,h and Supplementary Fig. 4). The effect on the Hill coefficient of 2GBI binding was also abolished (Fig. 5h and Supplementary Fig. 3). These findings indicate that the strengthening in allosteric coupling between subunits caused by the opposite charges at position 123 is not translated into stronger binding cooperativity when the binding sites are perturbed at position D112.

### Crosslinking the extracellular ends of S1 segments enhances allosteric coupling

We reasoned that, if D123 residues on adjacent open subunits were close enough to interact electrostatically with each other, the repulsive interaction between negative charges would be turned into an attractive interaction between a negative and a positive charge by the aspartate-to-arginine substitution in one subunit (WT-D123R dimer). The attractive interaction could strengthen the interface between the outer ends of S1 segments leading to a stronger allosteric coupling between subunits and to an increase in GBTA binding cooperativity.

To support this hypothesis, we searched for a way to strengthen the interface between the outer ends of S1 segments distinct from charge switching at position 123. Lee *et al.* had previously found that mutating Hv1 residue I127 to a cysteine results in spontaneous intersubunit cross-linking^17^. In addition, the crystal structure of the Hv1-CiVSP chimeric VSD showed that I127 is separated from the outer end of the S1 helix only by one residue^24^. So, we asked whether the formation of a covalent bond between cysteines at position 127, which is expected to produce a stronger S1-S1 interaction, would have an effect on GBTA binding cooperativity similar to that observed by altering the charges at position 123.

**Figure 6:**
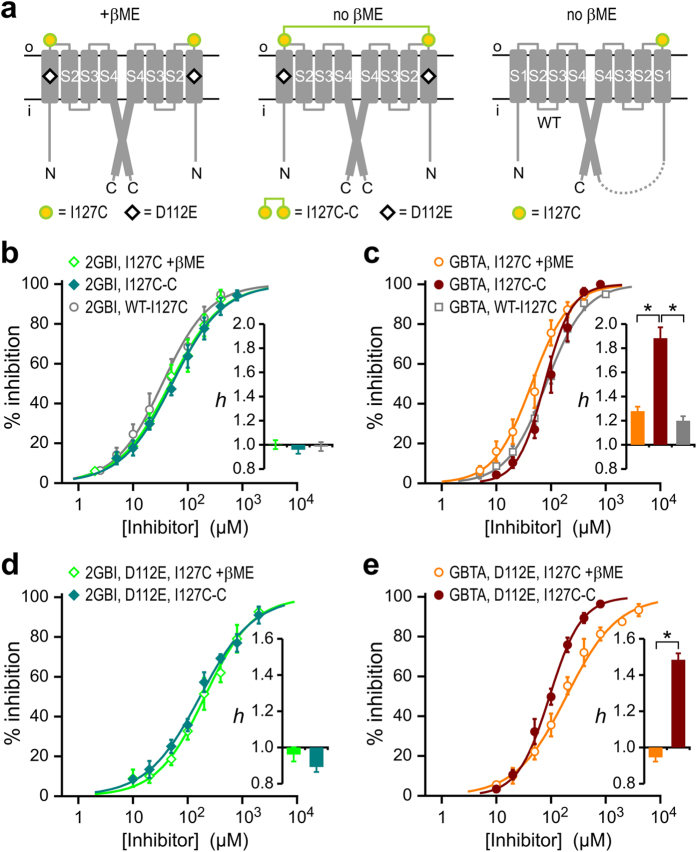
Open-state coupling is mediated by direct physical interaction between outer ends of S1 segments in the two subunits. (**a**) Schematics of mutant Hv1 dimers and linked dimers designed to study how intersubunit cysteine crosslinking affects GBTA binding cooperativity. (**b–d)** Concentration dependences of inhibition of the indicated constructs by 2GBI (**b**,**d**) and GBTA (**c**,**e**) in the presence or absence of 10 mM βME in the extracellular solution. Each point represents the average inhibition from 3 to 10 measurements ± S.D. Curve lines are Hill fits used to obtain K_d_ values (see Supplementary Table 1). Hill coefficients in inset histograms were determined from fits reported in Supplementary Figs 3 and 4. Asterisks in (**c**,**e**), indicate statistically significant differences between different conditions of GBTA inhibition (p < 0.0001, see methods). Corresponding differences for 2GBI inhibition [(**b**,**d**)] were not statistically significant (p > 0.05).

We measured concentration dependences for GBTA and 2GBI inhibition of Hv1 I127C in the presence and absence of 10 mM β-mercaptoethanol (βME) (Fig. 6a). While the inhibitors were added to the intracellular solution, the reducing agent was added to the extracellular solution. The linked dimer with the cysteine substitution in only one subunit (WT-I127C) was used as a negative control (Fig. 6a). We could not measure any effect of spontaneous intersubunit cross-linking between I127C subunits on 2GBI inhibition, as the Hill coefficients for binding to Hv1 I127C, with or without βME, were indistinguishable from the Hill coefficient for binding to the single-cysteine-substituted dimer WT-I127C (Fig. 6b and Supplementary Fig. 3). In stark contrast, we measured a large effect of spontaneous intersubunit cross-linking on GBTA inhibition. The Hill coefficient for binding to Hv1 I127C in the absence of βME (crosslink ON) was significantly higher than the Hill coefficient measured in the presence of βME (crosslink OFF), or the Hill coefficient measured with WT-127C linked dimer (crosslink absent) (Fig. 6c and Supplementary Fig. 4), indicating that the allosteric coupling between binding sites is considerably strengthened by the formation of the disulfide bond between outer ends of S1 segments. These results support our interpretation that an attractive electrostatic interaction between aspartate and arginine at positions 123 in the WT-D123R dimer is responsible for increased allosteric coupling between subunits.

We also found that the Hill coefficients of GBTA binding to the D112E I127C Hv1 dimer in the absence of βME was significantly higher than the Hill coefficient measured in the presence of the reducing agent (Fig. 6e and Supplementary Fig. 4), which means that the D112E mutation was not able to abolish the increase in cooperativity of GBTA binding produced by cysteine cross-linking at position 127. The Hill coefficients for 2GBI binding to the D112E, I127C Hv1 dimer were also not significantly affected by the D112E mutation (Fig. 6d and Supplementary Fig. 3).

Taken together, these findings show that strengthening the interaction between the outer ends of S1 segments in adjacent Hv1 subunits, either by attractive electrostatic interactions or by formation of a covalent bond between substituted cysteines, results in an increase in allosteric coupling between GBTA binding sites and resulting binding cooperativity. While the effect of the attractive electrostatic interaction on cooperativity can be abolished by mutation D112E, the effect of the covalent bond cannot.

## Discussion

In our exploration of the chemical space available for the binding of guanidine derivatives to the Hv1 channel, we found that 2-guanidinothiazoles like GBTA binds with a steeper concentration dependence than 2-guanidinobenzimidazoles (Fig. 1c). The analysis of the Hill coefficients for GBTA binding to dimeric and monomeric channels (Fig. 2a,b), and to dimeric channels in which one subunit was pre-bound to the inhibitor (Fig. 4) led us to conclude that Hv1 inhibition by GBTA is a cooperative process and that the binding sites for the compound in the two subunits are allosterically coupled. The finding that GBTA binds to open channels, as previously shown for the related compound 2GBI^32^, indicates that the allosteric coupling can be assessed specifically in the open state. The ability of our cooperative binding model to quantitatively describe GBTA inhibition of Hv1 (Fig. 2c) and to explain the different effects of 2GBI and GBTA on the decay of the channel’s tail current following membrane repolarization supports our interpretation of the binding process.

The maximal Hill coefficient that can be achieved in an allosteric protein with two binding sites like Hv1 is 2. We measured a coefficient of 1.31 for GBTA binding to Hv1 wild type, and the number increased to 1.88 in Hv1 I127C. The cooperative free energy 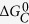, which is the difference between the binding free energies of the lowest affinity and highest affinity sites (see methods) was 1.3 kcal/mole in the case of Hv1 wild type and 2.7 kcal/mole in the case of Hv1 I127C. The binding of oxygen to hemoglobin is the best known and thoroughly studied example of a cooperative process^38^. For human hemoglobin (a tetramer with four allosterically coupled binding sites), the Hill coefficient is in the range 2.5–3.0, and the 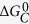 value varies from 1.26 to 3.64 kcal/mol, depending on experimental conditions^38^. So, in terms of global energetics, the cooperativity of GBTA binding to Hv1 is not substantially different from the binding of O_2_ to hemoglobin, when the different number of protein subunits in the two systems is taken into account.

**Figure 7:**
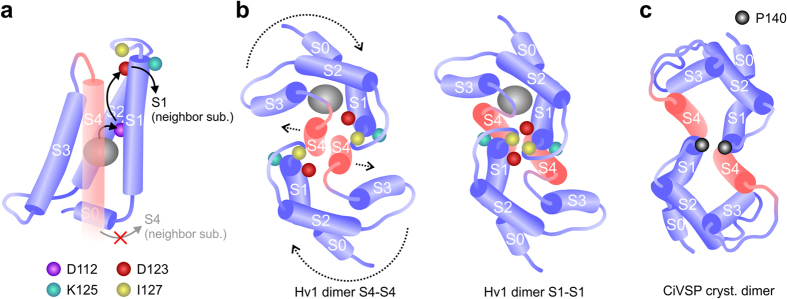
Dimeric organization of the channel’s open state explaining the observed intersubunit coupling. (**a**) Schematic representation of the Hv1 VSD. Helical segments are shown as cylindrical shapes based on the crystal structure of the Hv1-CiVSP chimera^24^. In the structure, the inner end of the S4 segment merges with the CCD (not shown). The predicted location of bound GBTA is shown as a gray oval shape. Black arrows indicate the pathway involved in allosteric coupling between the binding sites in the two neighboring subunits. Positions of the investigated S1 residues are marked by colored spheres. (**b**) Schematic representation of Hv1 subunits arranged in two distinct dimeric configurations, seen from the extracellular side of the membrane plane. On the left panel, the dimer interface is formed by the S4 helices^25^. 20-degree clockwise rotations of the two VSDs around axes perpendicular to membrane plane accompanied by a separation of the outer ends of the two S4 helices (dotted arrows) produce the arrangement shown on the right panel. In this configuration, residues D123 and I127 from neighboring subunits are allowed to get close together. (**c)** Schematic representation of CiVSP VSDs in the dimeric configuration found in the 4G80 crystal structure^44^. Position P140 in CiVSP corresponds to position D123 in Hv1.

In our cooperativity model, the binding of a GBTA molecule to one subunit causes an increase in binding affinity of the neighboring subunit. We envision a process in which a rearrangement of the binding environment produced by the first binding event (induced fit) leads to changes in intersubunit interactions. In response to these changes, the neighboring subunit alters its binding site resulting in tighter GBTA binding. In this process, S1 aspartate D112 is responsible for the rearrangements of the binding sites associated with the increase in binding affinity. D112 was previously shown to be part of the Hv1 proton permeation pathway and to act as selectivity filter^27^,^28^. Our results suggest that the selectivity filters in the two Hv1 subunits are allosterically coupled in the open state. Figure 7a shows the approximate location of the GBTA binding site and the positions of residues D112, D123, K125 and I127 on a schematic representation of the Hv1 VSD based on the crystal structure of the Hv1-CiVSP chimera^24^. The proposed direction of allosteric coupling involving the extracellular end of S1 is shown with black arrows.

Our findings indicate that a repulsive electrostatic interaction between residues at position 123 (123D/123D or 123R/123R) is associated with “normal” level of GBTA binding cooperativity in the open state, and that switching the interaction from repulsive to attractive (123D/123R), increases cooperativity (Fig. 5g). It could be expected that removing the repulsive interaction with an alanine substitution would also result in an increase in cooperativity. However, what is observed is a reduction in cooperativity in the 123A/123A dimer (Fig. 5e). One possible explanation is that the destabilizing effect of placing a hydrophobic residue in a hydrophilic environment may surpass the stabilizing effect deriving from the elimination of the repulsive interaction between D123 residues resulting in an overall decrease in binding cooperativity. Mony *et al.*^39^ have recently reported that solvent accessibility of position D171 of *Ciona intestinalis* Ci-Hv1 (which corresponds to D123 in human Hv1) increases upon activation, supporting the idea that D123 is located in a hydrophilic environment in the open state.

The gating of the Hv1 channel is known to occur through multiple transitions^19^,^20^,^26^,^39^,^40^,^41^. Qiu *et al.*^26^ found that, upon membrane depolarization, the voltage sensors of Ci-Hv1 undergo a conformational change that brings the channel in an activated -but still closed- state, followed by a distinct transition that leads to the opening of the proton conduction pathways in the two subunits. The conformational changes of the voltage sensors were monitored by voltage-clamp fluorometry^42^ and the second transition was found to be selectively perturbed by mutations at position D171. The perturbation of the fluorescence signal was consistent with the existence of an electrostatic interactions between D171 residues of adjacent subunits at some point along the reaction coordinate of the conformational change^26^. This interpretation is in agreement with our finding that D123 residues interact electrostatically in the open state and mediate allosteric coupling between GBTA binding sites.

Fujiwara *et al.*^25^ have proposed that the dimer interface at the cytoplasmic coiled-coil domain extends into the membrane to encompass the two S4 helices (Fig. 7b, left panel). This model of intersubunit interaction was based on a cysteine cross-linking analysis covering the entire VSD, and on a functional analysis of the region connecting the S4 helix to the CCD. In the absence of a transmembrane pH gradient, the Hv1 channel requires a rather strong membrane depolarization to open, and cysteine cross-linking is carried out under conditions in which the channel is mainly closed. Hence, the probed S4-S4 interface is likely to reflect the subunit configuration of the closed state. The fact that other studies have found evidence for the participation of S1 and S2 in intersubunit interactions during gating^17^,^21^,^26^ suggests that the channel may adopt different subunit configurations in the open and closed states, an idea consistent with the finding by Mony *et al.* that S1 moves during gating^39^.

Here we find that, in the open state, the extracellular ends of the S1 helices are in sufficiently close proximity to support direct electrostatic interactions that mediate allosteric coupling between subunits. In the dimer configuration with extended S4-S4 interactions, the S1 helices are too far from each other to interact directly. However, a 20-degree clockwise rotation of the two VSD subunits around axes perpendicular to the membrane plane combined with a separation of the outer ends of the two S4 helices produce an S1-S1 configuration that is consistent with our findings (Fig. 7b, right panel). We propose that the channel assumes this configuration in the open state.

While the CiVSP enzyme is believed to function as a monomer^43^, the crystal structure of its isolated VSD was captured in a dimeric state^44^. The outer ends of the S1 helices in this dimer are close in space, and the overall configuration resembles the one we propose for Hv1 (Fig. 7c). In the CiVSP dimer, the closest residues from adjacent subunits are prolines at position 140 (Fig. 7c). Interestingly, P140 in CiVSP corresponds to position D123 in Hv1. The similarities in subunit configuration between Hv1 and CiVSP dimers suggest that the VSDs of these proteins have an intrinsic tendency to form an interface in which the extracellular ends of S1 interact with each other.

The important role of Hv1 in sperm cell activation makes this channel an attractive drug target for controlling male fertility^9^. In addition, activation of Hv1 in microglia has been shown to worsen recovery from ischemic stroke^8^. Enhanced Hv1 activity was found to correlate with lower survival in patients with breast cancer^12^, or colorectal cancer^13^, and was proposed to contribute to B-cell malignancies^11^. Hence, small-molecule drugs targeting Hv1 could find applications as neuroprotective agents or anticancer therapeutics. The finding that guanidinothiazole derivatives can induce conformational rearrangements in open Hv1 subunits, which result in an increase in binding affinity, could lead to the development of more effective drugs targeting the Hv1 channel.

## Methods

### Channel constructs and expression

Site-directed mutagenesis on human Hv1 was performed using standard PCR techniques. In the Hv1NC_CiVSP_ construct, residues 1–96 and 228–273 of Hv1 were replaced by residues 1–113 and 240–576 of CiVSP^18^. In the Hv1 linked dimers, the C-terminus of one subunit was linked to the N-terminus of a second subunit through a GGSGGSGGSGGSGGSGG linker^18^. pGEMHE plasmids containing the various constructs were linearized with Nhe1 or Sph1 restriction enzymes (New England Biolabs), and RNA synthesis was carried out with a T7 mMessage mMachine transcription kit (Ambion). cRNAs were injected in *Xenopus* oocytes (50 nl per cell, 0.3–1.5 μg/μl) 1–3 days before the electrophysiological measurements. Stage V and VI oocytes from *Xenopus laevis* (NASCO) were obtained from Ecocyte Bioscience. After RNA injection, cells were kept at 18 °C in ND96 medium containing 96 mM NaCl, 2 mM KCl, 1.8 mM CaCl_2_, 1 mM MgCl_2_, 10 mM HEPES, 5 mM pyruvate, 100 μg/ml gentamycin, pH 7.2.

### Tested compounds

2-Guanidino-benzimidazole [**1**], 2-guanidino-benzothiazole [**2**], (4-methyl-1,3-thiazol-2-yl)guanidine [**5**], (5-bromo-4-methyl-1,3-thiazol-2-yl)guanidine) [**6**], ethyl 2-guanidino-5-methyl-1,3-thiazole-4-carboxylate [**8**], ethyl 2-guanidino-4-methyl-1,3-thiazole-5-carboxylate [**9**], and ethyl (2-guanidino-4-methyl-1,3-thiazol-5-yl)acetate [10], were from Sigma-Aldrich. Famotidine [**7**] was from MP Biomedicals. 1-[4-(4-chlorophenyl)-1,3-thiazol-2-yl]guanidine [**11**], and 1-[4-(3,4-dimethoxyphenyl)-1,3-thiazol-2-yl]guanidine [**12**] were from Matrix Scientific. These compounds were of the highest purity commercially available. They were dissolved in anhydrous DMSO to produce 100 mM stock solutions, which were then diluted in recording solution at the desired final concentration. Compounds 3 and 4 were synthesized as follows and were at least 99% pure.

#### (5-Trifluoromethyl-1,3benzothiazol-2-yl)guanidine [3] (CAS 159734-00-6)

To a suspension of 2-amino-4-(trifluoromethyl)benzenethiol hydrochloride (1.02 g, 4.5 mmol) in 25 mL aqueous hydrochloric acid (2.5 N) was added solid dicyandiamide (380 mg, 4.5 mmol) and the resulting heterogeneous mixture was refluxed for 4 hrs under vigorous stirring. The reaction mixture was allowed to cool to room temperature and was neutralized by gradual addition of 10 N potassium hydroxide. The white precipitate that formed was filtered, washed with cold water (3 × 50 ml), dried in an oven (65 °C) for several hrs and was recrystallized from ethyl acetate/petroleum ether to obtain a white solid (500 mg, 48%); m.p. 221–222 °C (Lit. 225–226 °C)^45^; ^1^H NMR (500 MHz, DMSO-d6): δ [ppm] = 7.25 (very broad s, 4 H), 7.40 (d, 1 H, J = 8.1 Hz), 7.73 (s, 1 H), 7.92 (d, 1 H, J = 8.1 Hz). ^13^C NMR (200 MHz, DMSO-d6): δ = 114.2 (d, J = 3.5 Hz), 117.5 (d, J = 3.5 Hz), 121.7, 124.6 (q, J = 272 Hz), 126.1 (q, J = 31.6 Hz), 134.8, 152.1, 158.4, 175.5. HRMS (ESI): *m/z* calcd. for C_9_H_8_F_3_N_4_S (M + H)^+^: 261.0416, found: 261.0419.

#### Naphtho[1,2-*d*][1,3]thiazol-2-yl-guanidine [4]

Naphtho[1,2-*d*]thiazol-2-amine (300 mg, 1.5 mmol), which was synthesized as previously described^46^, was heated in a small test tube to 200 ^°^C in an oil bath. 300 mg (large excess) of cyanamide and 1.0 ml of conc. hydrochloric acid were added rapidly to the hot compound, and the mixture was kept in the oil bath for about 2 minutes during which time most of the water evaporated. The reaction mixture was then allowed to cool down to room temperature and the resulting solidified material was broken into small chunks and washed with water to provide a slightly yellowish amorphous solid. (38 mg, 10%) m.p. 246-250 °C; ^1^H NMR (500 MHz, DMSO-d6, D_2_O): δ [ppm] = 7.59 (t, 1 H, J = 8.2 Hz), 7.66 (t, 1 H, J = 8.3 Hz),7.77 (d, 1 H, J = 8.6 Hz), 7.89 (d, 1 H, J = 8.6 Hz), 8.02 (d, 1 H, J = 8.2 Hz), 8.35 (d, 1 H, J = 8.3 Hz). ^13^C NMR (150 MHz, DMSO-d6): δ = 119.9, 122.7, 123.4, 123.6, 126.5, 127.1, 128.7, 132.1, 140.7, 169.1. HRMS (ESI): *m/z* calcd. for C_12_H_11_N_4_S (M + H)^+^: 243.0699, found: 243.0704.

### Electrophysiological measurements

Proton currents were measured in inside-out patches from oocytes expressing the different constructs using an Axopatch 200B amplifier controlled by pClamp10 software through an Axon Digidata 1440A (Molecular Devices). Intracellular and extracellular solutions had the same compositions: 100 mM 2-(N-morpholino)ethane-sulphonic acid (MES), 30 mM tetraethylammonium (TEA) methanesulfonate, 5 mM TEA chloride, 5 mM ethyleneglycol-bis(2-aminoethyl)-*N*,*N*,*N’*,*N’*-tetra-acetic acid (EGTA), adjusted to pH 6.0 with TEA hydroxide. All measurements were performed at 22 ± 2 °C. Pipettes had 1.5–4 MΩ access resistance. Current traces were filtered at 1 kHz, sampled at 5 kHz and analyzed with Clampfit10.2 (Molecular Devices) and Origin8.1 (OriginLab).

Solutions containing Hv1 inhibitors at various concentrations, and in some cases 10 mM βME, were introduced in the bath chamber under gravity via manifold connected to a VC‐6 perfusion valve system (Warner Instr.) controlled by the pClamp software via a TTL (Transistor-Transistor Logic) signal. Fast perfusion experiments were carried out with a multi‐barrel perfusion pencil (AutoMate Sci.) mounting a delivery tip 360 μm in diameter positioned in front of the patch pipette. Channel inhibition was determined by isochronal current measurements at the end of +120mV-depolarization pulses. G-V measurements were performed as previously described^18^,^20^. Unless otherwise specified, tail currents were recorded at −40 mV after depolarization steps at different voltages from −20 mV to +120 mV. A reference pulse preceding the test pulse was used to correct for current rundown^18^. G-V plots were fitted with the Boltzmann equation:


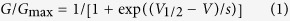


where V_1/2_ is the potential of half activation and *s* is the slope parameter, both expressed in mV.

### Analysis of inhibition curves

Apparent dissociation constants (K_d_) for the different combinations of channel and inhibitor (Supplementary Table 1) were determined by fitting the concentration dependences of inhibition (average %_inhib_ values) with the Hill equation:


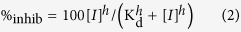


where [*I* ] is the concentration of inhibitor *I*, and *h* is the Hill coefficient. To calculate the Hill coefficients, equation (2) was rearranged to:


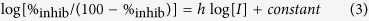


Inhibition data in the log-log form (Supplementary Figs 1, 3, and 4) were fitted by simple linear regression. When comparing *h* parameters obtained from equation (3) for two distinct channel-inhibitor pairs (*h*_*1*_ and *h*_*2*_ ), the statistical significance of their difference was evaluated by 2-tailed t-test. t-values were calculated using the equation:


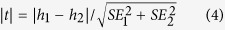


where *SE*_*1*_ and *SE*_*2*_ are the standard errors for *h*_*1*_ and *h*_*2*_ obtained from the corresponding linear regressions. To assess whether specific mutations affect binding cooperativity, Hill coefficients for 2GBI or GBTA inhibition of Hv1 mutants were individually compared to wild type. A total of 14 mutants were analyzed. To minimize the possibility of false positives, Bonferroni correction^47^ was applied to the level of statistical significance and the threshold p-value was set at 0.05/14.

### Cooperativity model of GBTA binding to the Hv1 channel

The cooperativity model used to describe Hv1 inhibition by GBTA (Fig. 2d) consists of two consecutive binding events:


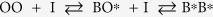


The block of one subunit by the first molecule of inhibitor causes an increase in binding affinity of the adjacent subunit in the dimer (BO* species). The dissociation constant for the first binding event is K_d1_ = [OO][I]/[BO*], and for the second binding event is K_d2_ = [BO*][I]/[B*B*], with K_d2_ < K_d1_. The percentage of inhibition can be calculated by equation (5):


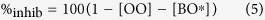


where [BO*] and [OO] are fractions of channels in the indicated conductive states (Fig. 2d). It is assumed that a channel in the OO state contributes twice as much current as a channel in the BO* state. By using the relationships between channel fractions established by the dissociation constants and the normalization equation: [OO] + 2[BO*] + [B*B*] = 1 in combination with equation (5), the following relationship between %_inhib_ and inhibitor concentration [*I*] can be derived:


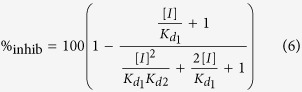


The concentration dependence of Hv1 inhibition by GBTA was fitted by equation (6) to determine the parameters K_d1_ and K_d2_ (Fig. 2c). In this model, cooperativity is quantified by the difference in free energy between the two binding events: 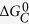 = *RT* log(K_d1_/K_d2_) (cooperative free energy). For positive cooperativity (*h* > 1), 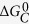 > 0. When K_d1_ = K_d2_ (no cooperativity, 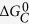 = 0), equation (6) becomes equation (2) with *h* = 1, which properly describes the binding behavior of 2GBI and GBTA to monomeric Hv1, and provides a reasonable approximation for the binding of 2GBI to dimeric Hv1. Equation (6) can also be used to describe negative binding cooperativity (*h* < 1) when K_d2_ > K_d1_ and 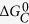 < 0.

### Assembly model for Hv1 subunits in the open state

The positions and orientations of the helical segments in individual VSDs within the Hv1 dimer model were based on the crystal structure of the Hv1-CiVSP chimera [3WKV]^24^. In the assembly shown on the left panel of Fig. 7b, the two VSDs contact each other along the S4 helix. This is the result of having the S4 and CCD helices merged together without significant breaking in secondary structure^25^, and the CCD in dimeric configuration, as observed in crystal structures of the isolated domain^22^,^23^. In the assembly shown on the right panel of Fig. 7b, the VSDs contact each other at the extracellular ends of the S1 helices. To achieve this assembly, the S4 segments were moved away from the central symmetry axis on the extracellular side, while maintaining the connection to the CCD on the intracellular side. The VSDs were also partially rotated to allow the S1 segments to get closer together.

## Additional Information

**How to cite this article**: Hong, L. *et al.* Interrogation of the intersubunit interface of the open Hv1 proton channel with a probe of allosteric coupling. *Sci. Rep.* **5**, 14077; doi: 10.1038/srep14077 (2015).

## Supplementary Material

Supplementary Information
