# Supplementary material for: Interrogation of the intersubunit interface of the open Hv1 proton channel with a probe of allosteric coupling
Source: Sci Rep. 2015 Sep 14;5:14077. doi: 10.1038/srep14077 (PMC4568520; doi:10.1038/srep14077)
Supplement: Supplementary Information [file srep14077-s1.pdf]

SUPPLEMENTARY INFORMATION

Interrogation of the intersubunit interface of the open Hv1 proton channel with a probe of allosteric coupling

Liang Hong, Vikrant Singh, Heike Wulff, and Francesco Tombola

Supplementary Text

Percentages of Hv1 inhibition reported in Fig. 1b were determined by measuring proton currents in inside-out patches from *Xenopus* oocytes expressing the human channel before and after addition of individual compounds to the intracellular solution at a concentration of 200 µM (Fig. 1b). We found that the addition of a trifluoromethyl group to the phenyl-ring of GBTA moderately increased potency (Fig. 1, compound [3]). On the other hand, removal of the phenyl-ring reduced activity (Fig. 1, compound [5]), an effect which could be compensated by introduction of various substituents onto the thiazole ring (Fig.1, compound [6]), suggesting that higher affinity compounds could be found among thiazole derivatives lacking the annulated phenyl ring. We tested this idea with compounds [7] through [12], and found that addition of a chlorophenyl group to the thiazole ring (compound [11]) resulted in a significant improvement in efficacy of inhibition compared to the reference compounds 2GBI and GBTA (Fig. 1b-c).

Supplementary Results

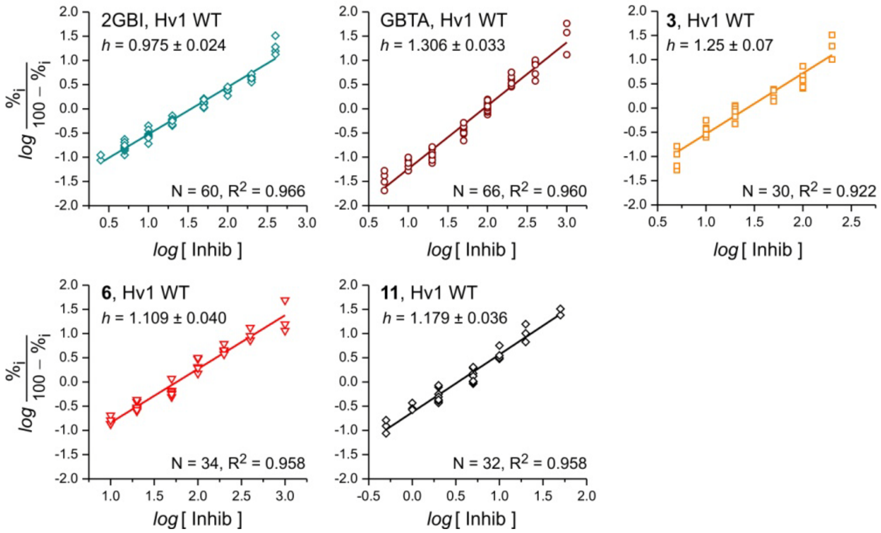

Supplementary Fig. 1. Log-log graphs of the concentration response curves of Hv1 inhibition by four 2-guanidinothiazoles compared to 2GBI. Data points were fitted by simple linear regressions. Hill coefficients (h) are the slopes of the linear fits (equation (3), in Methods section). The h values reported here are also found in the legend of Fig. 1c. Number of points (N) and adjusted R^2 value are provided for each fit.

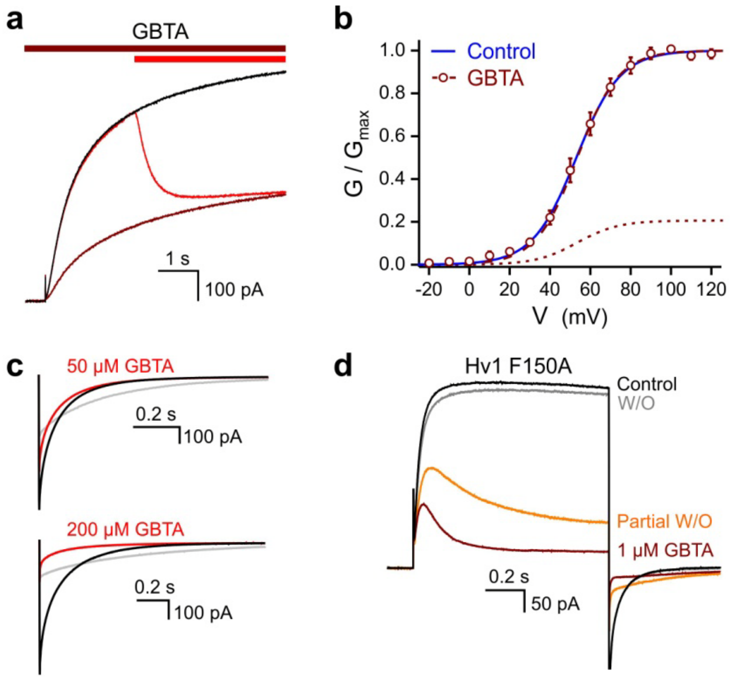

Supplementary Fig. 2. Characteristics of Hv1 block by GBTA. a) GBTA blocks open Hv1 channels. Proton currents from Hv1 wild type elicited by a depolarization step from -80 mV to +120 mV and measured in the absence of the inhibitor (black trace), or in the presence of GBTA (100 μM) (red and dark-red traces). $pH_i = pH_o = 6.0$. Horizontal bars indicate presence of the inhibitor in the intracellular solution. **b)** GBTA does not inhibit Hv1 by hindering channel opening. G-V curves of wild type Hv1 measured in the absence (blue) and in the presence of 200 μM GBTA (dark red). Error bars on data points are S.E.M. Boltzmann fit is shown as dashed line. Parameters are $V_{1/2} = 53.6 \pm 2.3$, slope = 10.3 ± 0.6, n=4. Only Boltzmann curve is shown for the control (blue). See ref. ([19]) for parameters. Dotted line is the G-V in the presence of the inhibitor accounting for the reduction in maximal conductance. **c)** Effect of GBTA on tail currents of Hv1 wild type differs from 2GBI. Tail currents were measured at -80 mV after a depolarization step at +120 mV (not shown). $pH_i = pH_o = 6.0$. In the presence of intracellular GBTA, the current is reduced but the decay is not significantly slowed down (negligible cross-over between red and black traces). Light-gray curves indicate tail currents expected in case the blocker behaved like 2GBI. **d)** Inhibition of Hv1 F150A by GBTA. Currents were elicited by a depolarization step from -80 mV to +120 mV, $pH_i = pH_o = 6.0$. Current traces measured in the absence of the inhibitor and in the presence of 1 μM GBTA are in black and dark red, respectively. Orange and gray traces were measured after partial and complete inhibitor washout (W/O). As previously shown for 2GBI, the channel needs to open in order for GBTA to block the current during the depolarization step. GBTA slows down the decay of the tail currents measured after the depolarization pulse in Hv1 F150A, but to a lower extent compared to 2GBI.

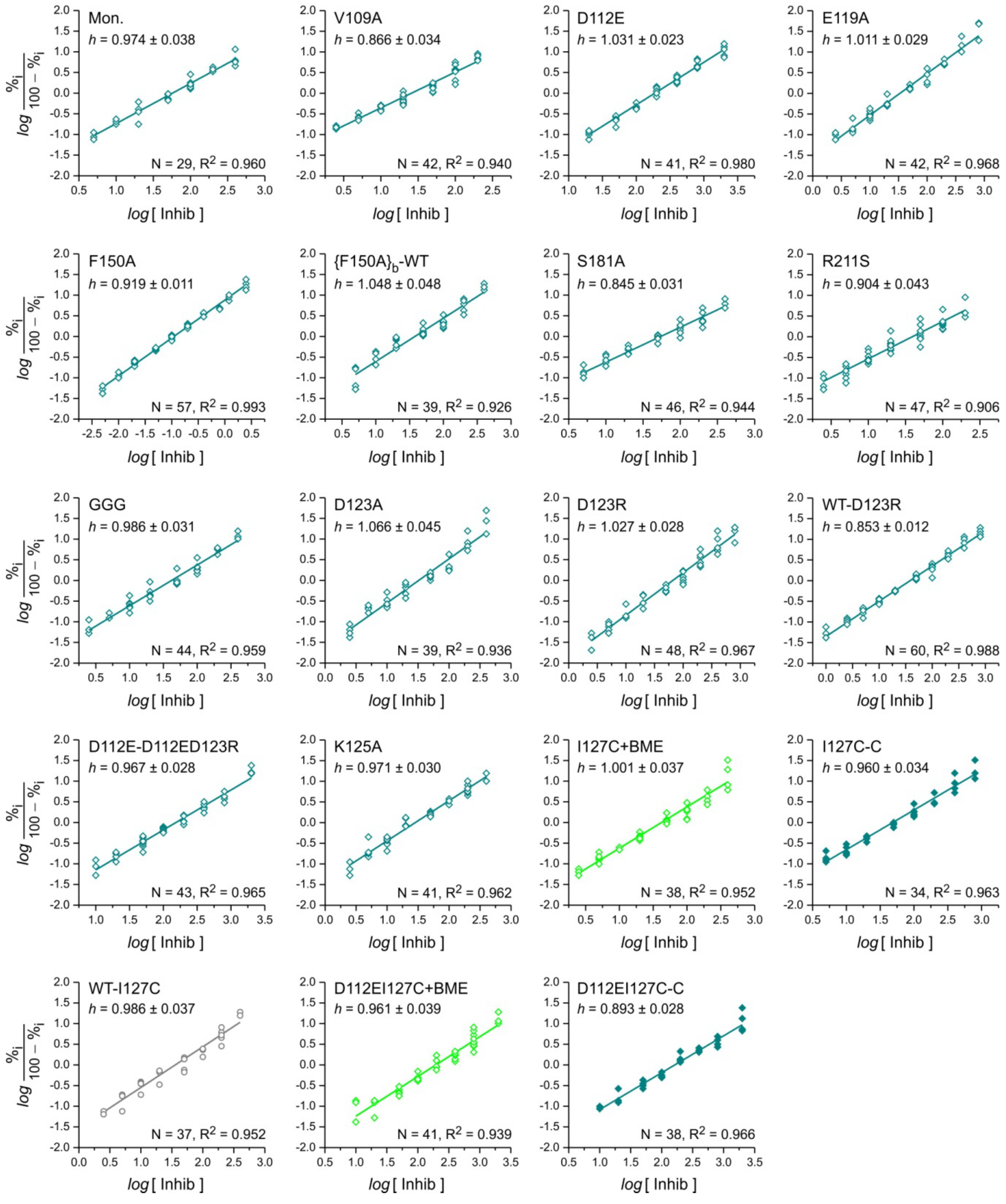

Supplementary Fig. 3. Log-log graphs of the concentration response curves of 2GBI inhibition for the indicated mutant channels. Data points were fitted by simple linear regressions (equation (3), in Methods section). Slopes of the linear fits (Hill coefficients *h*) are reported in the histograms of Figures 2 to 6. Number of points (N) and adjusted R^2 value are provided for each fit.

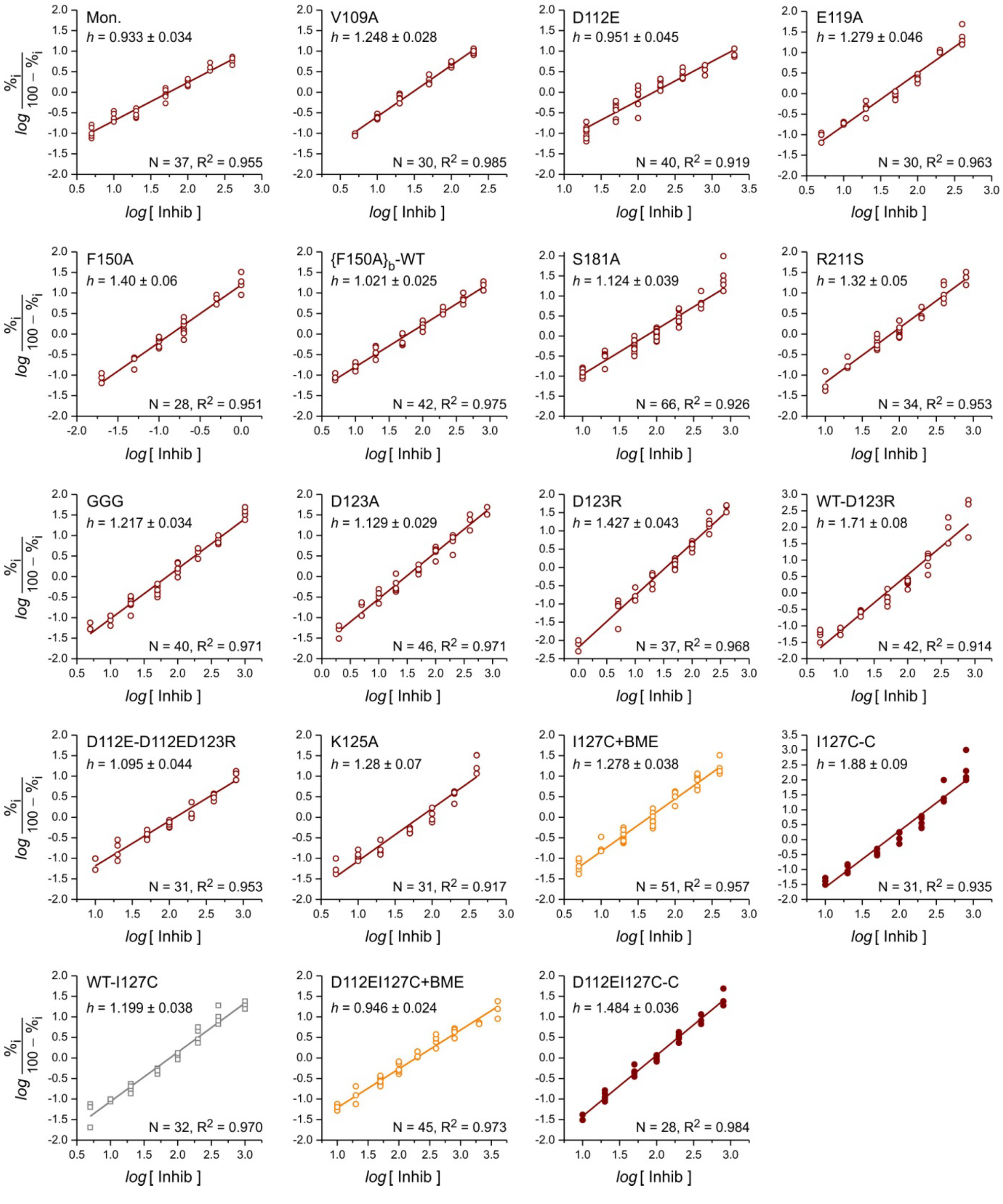

Supplementary Fig. 4. Log-log graphs of the concentration response curves of GBTA inhibition for the indicated mutant channels. Data points were fitted by simple linear regressions (equation (3), in Methods section). Slopes of the linear fits (Hill coefficients h) are reported in the histograms of Figures 2 to 6. Number of points (N) and adjusted R^2 value are provided for each fit.

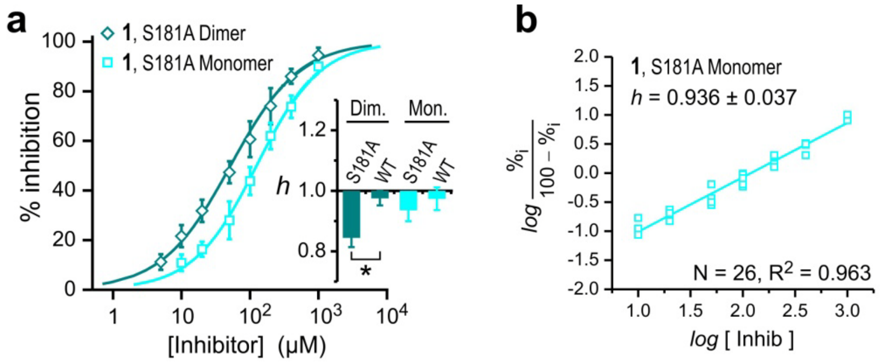

Supplementary Fig. 5. Effects of mutation S181A on 2GBI binding to dimeric and monomeric Hv1. a) Concentration dependences of inhibition of dimeric (teal) and monomeric (cyan) Hv1 S181A by 2GBI. Each point represents the average inhibition from 3 to 9 measurements ± S.D. Curve lines are Hill fits used to obtain apparent K_d values (see Supplementary Table 1). Hill coefficients (h) shown in inset histogram were determined from fits reported in (b) and Supplementary Figs. 1 & 3. The difference in Hill coefficient between mutant and WT channel is statistically significant only in the dimer background ($p<0.05/14$). **b)** Log-log graphs of the concentration response curve of 2GBI inhibition for monomeric Hv1 S181A. Data points were fitted by simple linear regressions (equation (3), in Methods section). Slope of the linear fit is h.

Supplementary Table 1. Apparent K_d values from Hill fits of concentration dependences of channel inhibition

n	Channel / Inhibitor	K_d (μM) (±SE)
1	Hv1 WT / 2GBI	38 ± 6*
2	Hv1 WT / GBTA	90 ± 7
3	Hv1 WT / [3]	24.7 ± 2.6
4	Hv1 WT / [6]	57.4 ± 2.3
5	Hv1 WT / [11]	3.04 ± 0.04
6	$Hv1NC_{CiVSP}$ (monomer) / 2GBI	56.1 ± 2.4
7	$Hv1NC_{CiVSP}$ (monomer) / GBTA	54.2 ± 3.5
8	V109A / 2GBI	22.1 ± 1.6*
9	V109A / GBTA	31.1 ± 1.2
10	D112E / 2GBI	191 ± 8*
11	D112E / GBTA	159 ± 18
12	F150A / 2GBI	0.118 ± 0.007*
13	F150A / GBTA	0.133 ± 0.007
14	S181A / 2GBI	51.9 ± 2.4*
15	S181A / GBTA	71 ± 6
16	S181A $Hv1NC_{CiVSP}$ (monomer) / 2GBI	125 ± 6
17	R211S / 2GBI	41.3 ± 2.1*
18	R211S / GBTA	77.7 ± 3.9
19	$\{F150A\}_b$-WT / 2GBI	35.4 ± 3.6
20	$\{F150A\}_b$-WT / GBTA	63.2 ± 3.3
21	GGG / 2GBI	44.5 ± 2.5
22	GGG / GBTA	74.4 ± 4.8
23	E119A / 2GBI	34.4 ± 2.9
24	E119A / GBTA	40.5 ± 2.9
25	D123A / 2GBI	35.3 ± 3.5
26	D123A / GBTA	31.6 ± 1.5
27	D123R / 2GBI	71.5 ± 4.1
28	D123R / GBTA	35.8 ± 2.4
29	WT-D123R / 2GBI	40.0 ± 0.7
30	WT-D123R / GBTA	55.0 ± 4.1
31	D112E-D112E/D123R / 2GBI	146 ± 10
32	D112E-D112E/D123R / GBTA	136 ± 9
33	K125A / 2GBI	29.4 ± 1.7
34	K125A / GBTA	86 ± 6
35	I127C+βME / 2GBI	47.6 ± 3.5
36	I127C-C / 2GBI	51.7 ± 2.2
37	WT-127C / 2GBI	34.4 ± 2.3
38	I127C+βME / GBTA	44.3 ± 2.5
39	I127C-C / GBTA	76 ± 7
40	WT-127C / GBTA	81.5 ± 3.7
41	D112E I127C+βME / 2GBI	206 ± 13
42	D112E I127C-C / 2GBI	168 ± 9
43	D112E I127C+βME / GBTA	191 ± 11
44	D112E I127C-C / GBTA	94.2 ± 2.8

*Values are the same as reported in ref. ([29]).
